# Antiparasitic Efficacy of Curcumin Against *Besnoitia besnoiti* Tachyzoites *in vitro*

**DOI:** 10.3389/fvets.2018.00333

**Published:** 2019-01-11

**Authors:** María Eugenia Cervantes-Valencia, Carlos Hermosilla, Yazmín Alcalá-Canto, Graciela Tapia, Anja Taubert, Liliana M. R. Silva

**Affiliations:** ^1^Graduate Program of Animal Health and Production, Facultad de Medicina Veterinaria y Zootecnia, Universidad Nacional Autónoma de México, Mexico City, Mexico; ^2^Institute of Parasitology, Justus Liebig University Giessen, Giessen, Germany; ^3^Department of Parasitology, Facultad de Medicina Veterinaria y Zootecnia, Universidad Nacional Autónoma de México, Mexico City, Mexico; ^4^Department of Genetics and Biostatistics, Facultad de Medicina Veterinaria y Zootecnia, Universidad Nacional Autónoma de México, Mexico City, Mexico

**Keywords:** curcumin, *Besnoitia besnoiti*, bovine besnoitiosis, antiparasitic effect, *in vitro*, tachyzoite

## Abstract

*Besnoitia besnoiti* is the causative agent of bovine besnoitiosis. *B. besnoiti* infections lead to reduced fertility and productivity in cattle causing high economic losses, not only in Europe, but also in Asia and Africa. Mild to severe clinical signs, such as anasarca, oedema, orchitis, hyperkeratosis, and characteristic skin and mucosal cysts, are due to *B. besnoiti* tachyzoite and bradyzoite replication in intermediate host tissues. So far, there are no commercially available effective drugs against this parasite. Curcumin, a polyphenolic compound from *Curcuma longa* rhizome is well-known for its antioxidant, anti-inflammatory, immunomodulatory and also anti-protozoan effects. Hence, the objective of this study was to evaluate the effects of curcumin on viability, motility, invasive capacity, and proliferation of *B. besnoiti* tachyzoites replicating in primary bovine umbilical vein endothelial cells (BUVEC) *in vitro*. Functional inhibition assays revealed that curcumin treatments reduce tachyzoite viability and induce lethal effects in up to 57% of tachyzoites (IC_50_ in 5.93 μM). Referring to general motility, significant dose-dependent effects of curcumin treatments were observed. Interestingly, curcumin treatments only dampened helical gliding and twirling activities whilst longitudinal gliding motility was not significantly affected. In addition, curcumin pretreatments of tachyzoites resulted in a dose-dependent reduction of host cell invasion as detected by infections rates at 1 day p. i. These findings demonstrate feeding cattle with *Curcuma longa* rhizomes may represent a new strategy for besnoitiosis treatment.

## Introduction

*Besnoitia besnoiti* (phylum Alveaola, subphylum Apicomplexa, family Sarcocystidae) is an intracellular obligate parasite infecting cattle phylogenetically closely related to *Toxoplasma gondii* and *Neospora caninum* (1, 2).

Bovine besnoitiosis, which is an emergent disease in Europe but is also vastly endemic in Asia and Africa, causes considerable economic losses in cattle industry (1). Even though a high percentage of *B. besnoiti-*infected animals are commonly asymptomatic, some of them show mild to severe disease. Thus, during the acute phase of bovine besnoitiosis, fast replication of tachyzoites in host endothelial cells of different organs and vessels causes severe symptoms such as fever, nose and eye discharge, photophobia and anasarca, accepting a variable clinical prevalence of between 1 and 10% in endemic herds. Nevertheless, the mortality rate depends on whether besnoitiosis is endemic or epidemic (2). During the chronic phase of infection, slow replicating bradyzoites induce the formation of large-sized thick-walled cysts mainly found in subcutaneous tissues and mucous membranes (1). Furthermore, decline in milk, and infertility (temporary or permanent) in bulls due to orchitis are observed (3). Presently, the complete life cycle of *B*. *besnoiti* is not entirely solved and especially final host species are still unknown. Nevertheless, direct contact between infected and non-infected animals (e.g., natural mating, naso-pharyngeal route) and insect-mediated transmission (i.e., tabanids, *Stomoxys calcitrans*) (2, 4) have been suggested as suitable transmission routes. Hence, to prevent and avoid rapid spreading of the disease in Europe, effective treatment and diagnostic measures are urgently needed. So far, diagnosis of cattle besnoitiosis is mainly based on the occurrence of typical cystic lesions on scleral conjunctiva or dermis and on serological tools (i.e., ELISA and western blot) (2, 5).

So far, no effective treatment against *Besnoitia* spp. is commercially available (1). In the last years, several drugs such as nitazoxanide, tizoxanide, sulfadiazine, thiazolides, biphenylimidazoazines, bumped kinase inhibitors (BKIs), diclazuril, decoquinato, and naphto-quinone buparvaquone have been tested for their efficacy against *Besnoitia* spp. (6–11). Furthermore, no vaccines against cattle besnoitiosis are licensed in Europe (2), even though the usage of recently tested live and attenuated vaccines could increase the risk of introducing the parasite into non-infected herds and of having carrier animals in the herds (1). Consequently, there is an urgent need for alternative control measures for bovine besnoitiosis.

Curcumin is a polyphenol present in the *Curcuma longa* rhizome and it has been widely used for centuries both as a spice or in traditional medicine based on its well-known anti-oxidant and anti-inflammatory properties (12). More recently, several investigations have proven that curcumin also exhibits anti-parasitic effects (13). In times of increasing resistance of several parasites against synthetic antiparasitic drugs worldwide, which imposes a huge problem in livestock production (14, 15), the application of bioactive plant compounds represent an important alternative measure to control parasitoses (16–18). Therefore, *in vitro* anti-protozoan effects of curcumin against numerous species have recently been described, i.e., *Eimeria tenella, E. bovis, Giardia intestinalis, Plasmodium falciparum, P. berghei, Leishmania* spp., *Trypanosoma cruzi, T. evansi, Cryptosporidium parvum, Neospora caninum*, and *Toxoplasma gondii* (19–26). In addition, *in vivo* effects of curcumin treatments were also documented in rabbit and sheep coccidiosis (27, 28). Overall, the molecular mechanism of curcumin action is not known so far, but interference with intracellular organelles and cytoskeleton or with cellular metabolism is postulated (29), which might impact on protozoan parasites. Furthermore, curcumin inhibits the glyoxalase system of *T. gondii* (24) and efficiently blocks histone deacetylation in curcumin-exposed *P. falciparum* (25).

The aim of the current study was to evaluate the effects of curcumin on the viability and motility of *B. besnoiti* tachyzoites, as well as to determine its impact on tachyzoite host cell invasion and intracellular parasite proliferation in primary bovine host endothelial cells *in vitro*.

## Materials and Methods

### Parasites

*B. besnoiti* (strain Eb1Evora04) was maintained by several passages of tachyzoites in primary bovine umbilical vein endothelial cells (BUVEC) according to previous reports (30). Freshly released tachyzoites were collected from cell culture supernatants, filtered with a 5 μm syringe filter (Sartorius®), centrifuged (350 × *g*, 12 min), counted (Neubauer chamber), and then suspended in modified endothelial cell growth medium [ModECGM; ECGM (PromoCell®) mixed with 70% (v/v) M199 (Sigma-Aldrich®), 10% fetal calf serum (FCS; Gibco®) and 1% penicillin-streptomycin (PS; Sigma-Aldrich®)] shortly before usage.

### Host Cells

Primary BUVEC were isolated according to Taubert et al. (31) and cultured in ModECGM. Cell culture medium was changed every 2 days until cells reached 80% confluency. To determine the influence of treatments on infection and proliferation capacities of parasites, different BUVEC isolates (*n* = 3, 12-well formats, Greiner) were cultured for 24 h and 48 h (37°C, 5% CO_2_) after *B. besnoiti* tachyzoite infection. BUVEC cell layers were used for infection after 2–3 passages *in vitro*.

### Effects of Curcumin on *B. Besnoiti* Tachyzoite Viability and Motility

For all *in vitro* studies, a stock solution of 20 mM curcumin (Sigma-Aldrich®, C1386) in DMSO (dimethyl sulfoxide, Sigma-Aldrich®) was prepared and used for different dilutions (1, 2, 4, and 8 μM) in ModECGM. Plain medium (ModECGM) and medium containing DMSO (DMSO, 1:2,500) were used as medium and solvent controls, respectively.

For viability-related experiments, 5 × 10^6^ tachyzoites were incubated for 90 min with increasing doses of curcumin (1, 2, 4, 8 μM; 5% CO_2_, 37°C). Viability of tachyzoites was determined by the trypan blue (Sigma-Aldrich®) staining assay (19). Unstained parasites were considered as viable.

Motility tests were performed according to a previous report on *T. gondii* tachyzoites (32). The same number of tachyzoites, doses of curcumin, and incubation time were used as in viability experiments. After incubation, tachyzoites were washed twice in plain medium to remove any traces of curcumin or DMSO. To assess the effects of curcumin on tachyzoites motility, five different vision power fields per treatment (50–60 tachyzoites per condition) were randomly recorded for 2 min each using an inverted microscope (IX81, Olympus®) equipped with a XM10 camera (Olympus®), in 12-well cell culture plates and analyzed later on. Different types of movements were observed and quantified: general motility (any movement), gliding motility (or longitudinal gliding), twirling and helical-gliding, as previously described (33).

### Cytotoxicity of Curcumin for Host Endothelial Cells

In order to determine if curcumin had cytotoxic effects on BUVEC, XTT cell viability assays (CyQUANT^TM^, Invitrogen) were performed. Therefore, a total of 5 × 10^3^ BUVEC/well were seeded (96 well-plate) and cultured at 37°C, 5% CO_2_ until confluency. Increasing concentrations of curcumin (0.25, 0.50, 1, 2, 4, 8, and 16 μM) were added to BUVEC and incubated for 90 min and 180 min. Thereafter, XTT cell viability assays were performed according to the manufacturer's instructions. Plain medium and DMSO-supplemented medium (1:2,500) were used as controls.

### Effects of Curcumin on *B. Besnoiti* Tachyzoite Host Cell Invasion and Intracellular Proliferation

Following pretreatments (90 min; 1–8 μM curcumin, 37°C, 5% CO_2_), *B. besnoiti* tachyzoites were washed twice in ModECGM to remove any curcumin/DMSO residues. Confluent BUVEC (*n* = 3) were infected with pre-treated tachyzoites (curcumin) or non-treated control parasites (ModECGM, DMSO) at a multiplicity of infection (MOI) of 1:4. At 24 h post infection (p. i.), infection rates were determined microscopically by counting infected and non-infected BUVEC in 16 visual power vision fields (400 x) per treatment. After 48 h p. i., freshly released tachyzoites were collected from cell culture supernatants, centrifuged (350 × *g*, 12 min) and counted using a Neubauer chamber to determine the proliferation capacity of pre-treated *B. besnoiti* tachyzoites.

### Statistical Analysis

IC_50_, IC_90_, and IC_99_ values (concentrations which inhibit 50, 90, and 99% tachyzoite survival) were calculated by probit regression analyses for viability assay. For motility/movement assays, a univariate analysis of variance was performed and multiple comparison tests were applied (Tukey or Dunn's, general motility and type of movement, respectively). Normal distribution of the data was confirmed for infection rate (%) data with Shapiro-Wilk Test (*p* > 0.05). The data were then analyzed by a general linear model with treatment [control, DMSO and curcumin (1, 2, 4, and 8 μM)] as fixed factor and BUVEC cells as random factor, with least square means method. Given that the data of tachyzoites production showed a Poisson distribution, they were analyzed by a generalized linear model (GzLM) with maximum-likelihood as method of estimation, applying link log and Poisson distribution. The marginal means were estimated for each treatment and compared by pairs of groups by Bonferroni test. Statistical analyses were performed using SPSS software (IBM SPSS® Statistics) version 21. Significant statistical differences were considered at *p* < 0.05.

## Results

### Curcumin Treatments Impair *B. Besnoiti* Tachyzoite Viability

Treatments with 1, 2, 4, and 8 μM curcumin led to an enhanced tachyzoite mortality rate of 21.3, 34.7, 38.7, and 57%, respectively (Table 1). In contrast, in medium and solvent controls, a mortality rate of 6 and 12% was observed, respectively. Overall, curcumin treatments resulted in dose-dependent lethal effects on *B. besnoiti* tachyzoites probit regression was significant at *p* = 0.0001 Zc = 9.31; IC_50_, IC_90_, and IC_99_ were 5.93, 112.59, and 1240.35 μM, respectively (Table 1). Upon curcumin exposure (90 min), tachyzoites lost their characteristic half-moon- or banana-shape form, presenting a blunt tip at the apical edge. In addition, the tachyzoite pellicula appeared affected and showed an irregular morphology (data not shown).

**Table 1:** Effects of curcumin treatments on *B. besnoiti* tachyzoite viability.

	**Dose**	**Mortality (%)**	**IC_**50**_ (μM)** **(LL, UL)**	**IC_**90**_ (μM)** **(LL, UL)**	**IC_**99**_ (μM)** **(LL, UL)**	**χ^2^** **(P)**
Medium control	–	6.50	5.93 (4.87, 7.75)	112.59 (58.22, 306.96)	1240.35 (425.16, 6372.65)	6.97 (0.72)
Solvent control	–	12.05				
Curcumin	1 μM	21.32				
	2 μM	34.72				
	4 μM	38.70				
	8 μM	56.97				

*IC, inhibitory concentration; LL, lower limit; UL, upper limit*.

### Curcumin Treatments Affect *B. Besnoiti* Tachyzoite Motility

**Figure 1:**
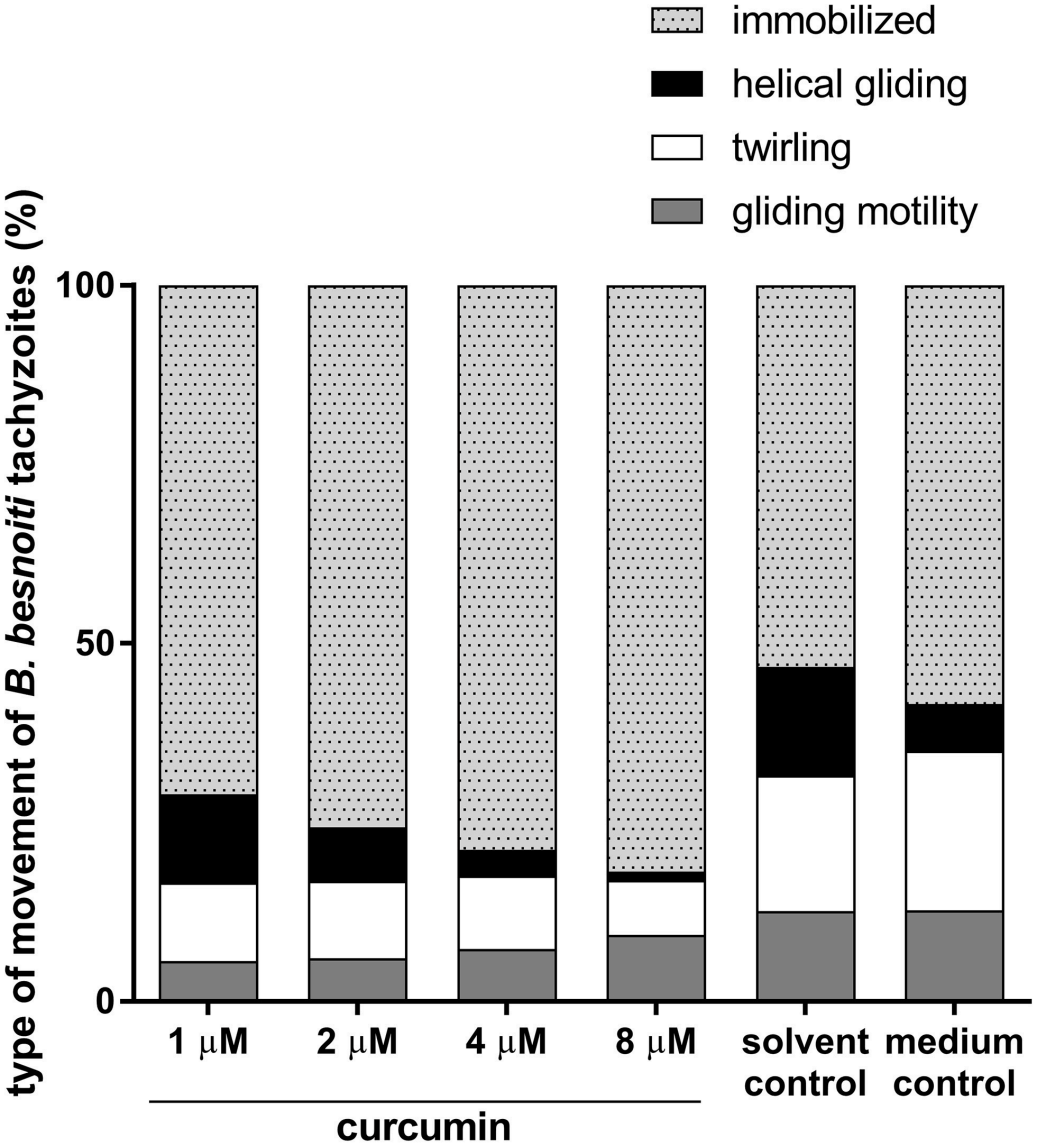
Effects of curcumin treatments on different types of *B. besnoiti* tachyzoites movement. *B. besnoiti* tachyzoites were treated with curcumin different doses (1–8 μM) and controlled microscopically for immobilization, helical gliding, twirling and gliding motility.

Tachyzoites were considered as immobilized when they failed to move during the entire observation period of 2 min. Overall, a significantly reduced general motility of tachyzoites was detected at curcumin concentrations of 4 μM (*p* = 0.027) and 8 μM (*p* = 0.0001) when compared to negative controls (please also refer to Video 1 in Supplementary Material). Broken down on the three different types of movement, twirling and helical gliding were significantly reduced at 8 μM curcumin treatments (*p* = 0.0159, *p* = 0.0135, respectively) whilst gliding motility was not significantly affected (Figure 1).

### Curcumin Pretreatments Block *B. Besnoiti* Tachyzoite Host Cell Invasion

**Figure 2:**
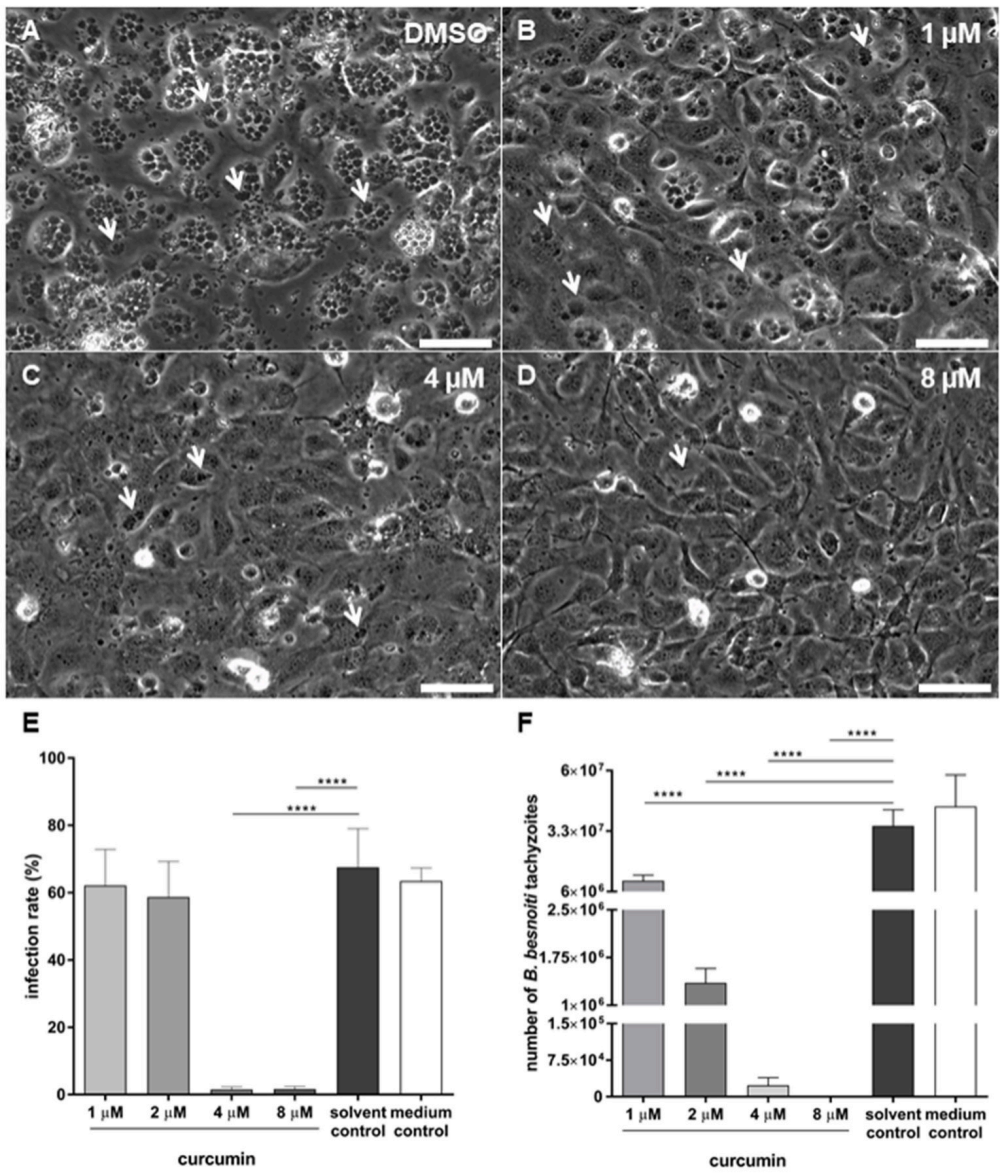
Effects of curcumin pretreatments of *Besnoitia besnoiti* tachyzoites on infection rate and parasite proliferation in bovine endothelial host cells. *B. besnoiti* tachyzoites were pretreated with different doses of curcumin (1, 2, 4, and 8 μM) for 90 min and used for bovine umbilical vein endothelial cell (BUVEC) infection [for illustrations, see **(A–D)**, bar 50 μm]. At 24 h p. i., infection rates were determined microscopically **(E)** and at 48 h p. i. tachyzoite production was analyzed **(F)** (^****^p < 0.0001).

As an obligate intracellular parasite, *B. besnoiti* needs to actively invade host endothelial cells *in vivo* in order to successfully replicate and to complete its life cycle. Infection rates were determined at 24 h p. i., when typical meront stages containing rosettes structures with newly formed tachyzoites (white arrows, Figure 2) are present in host cells. Overall, curcumin pretreatments of tachyzoites led to a significant reduction of infection rates in BUVEC (*p* < 0.0001) *in vitro* (Figure 2E). As such, medium- and solvent-treated tachyzoites invaded BUVEC at a high percentage and led to an infection rate of 63.5 and 67.5%. In contrast, host cell infections with curcumin-treated tachyzoites resulted in the following infection rates: 1 μM (62.10%), 2 μM (58.69%), 4 μM (1.48%), and 8 μM (1.61%). Significant differences were observed at 4 and 8 μM curcumin treatments (both, *p* < 0.0001). Additionally, cytotoxicity assay (XTT) revealed that concentrations higher than 8 μM curcumin (percentage of control) resulted in adverse effects on exposed BUVEC (please refer to Figure 1 in Supplementary Material; IC_50_ 6.3 μM). In all experiment settings, treated-tachyzoites were always washed after the incubation period (90 min), reducing significantly the amount of curcumin that the host cells could eventually being exposed to, confirming that inhibition of invasion was not due to any curcumin-derived cytotoxic effect.

### Curcumin Hampers *B. Besnoiti* Tachyzoites Replication in BUVEC

Curcumin treatments had a considerable effect on intracellular parasite proliferation. As such, a significant decrease of newly released *B. besnoiti* tachyzoite numbers was observed in curcumin-treated groups at 48 h p. i. when compared to non-treated controls (Figure 2F). As such, significant differences (all *p* < 0.0001) in tachyzoite replication were detected between the solvent control (3.53 × 10^7^ ± 7.23 × 10^6^) and 1 μM (1.09 × 10^7^ ± 2.51 × 10^6^), 2 μM (1.35 × 10^6^ ± 2.28 × 10^5^), 4 μM (2.31 × 10^4^ ± 1.63 × 10^4^), and 8 μM (0.00 ± 0.00) treatments. It appears obvious that less tachyzoites are produced if initial infection rates are lower (Figure 2E). However, given that infection rates were not affected in the case of 1 and 2 μM treatments (Figure 2E), the antiproliferative effects of curcumin at these doses must be related to compound-induced impairment of parasite replication thus proving that curcumin indeed impairs *B. besnoiti* intracellular replication in primary host endothelial cells.

## Discussion

Bovine besnoitiosis is spreading in Europe (34, 35), but also in other continents, such as Africa and Asia. Nonetheless, no commercial drugs against *B*. *besnoiti* are currently available; thus efforts on alternative effective control strategies are urgently needed to prevent further spread of this disease. In the recent years, several synthetized compounds have been tested against *B. besnoiti in vitro*, e.g., nitazoxanide, tizoxanide and sulfadiazine, thiazolides, biphenylimidazoazines, bumped kinase inhibitors (BKIs), diclazuril, decoquinato, and naphto-quinone buparvaquone (6–11). In a new *in vitro* study, well-known anti-coccidial drugs such as decoquinate and diclazuril showed to inhibit infection rates by 90 and 83% at 0 h p. i., respectively (14). Even though repurposing of commercially available anti-coccidial drugs is a reasonable strategy for identifying therapeutic compounds against *B. besnoiti*, during the last decades, the abuse and massive usage of anticoccidial drugs for control of other apicomplexan parasites (i.e., *Eimeria, Sarcocystis, Cystoisospora, Neospora*) has generated the development of parasite resistances against these commercial compounds. In general, anticoccidial drugs are known to hamper development of intracellular parasite stages (sporozoites, trophozoites, merozoites, gametocytes), and most of them can be administrated as food additives or diluted in drinking water. Starting in the 1960s and until now, resistance to anticoccidian drugs has been increasing and it has been reported to occur largely in chicken and swine industries due to inadequate or abuse drug usage (33, 36–38). Since 2006, the European Union (EU) has strictly limited the usage of chemical anticoccidian compounds as food/water additives even proposing an entire ban from 2021 onwards (Council Directive of 2011/50/EU of The European Council) to prevent residues of coccidiostatics in animal products (39). Therefore, usage of alternative bioactive plant compounds with natural anticoccidial efficacies represent a good alternative to synthetic drugs. So far, few herbal-based anticoccidial compounds are available on the market, especially to be used as food additives in poultry and other industries (13). Using anticoccidial bioactive plant compounds as food supplements will further facilitate the administration process and avoid extra animal handling for treatments.

Curcumin is a polyphenol from *C. longa* rhizome with antioxidant and antiparasitic effects. As such, *Eimeria-*infected animals showed diminished oocysts shedding and less lesions after curcumin treatment (19, 27, 40, 41). More importantly, daily weight gains revealed two-fold higher in curcumin-treated farm animals compared to non-treated controls (28). In addition, curcumin treatments also resulted in improved quality of meat products (prolonged storage stability), most probably due to antioxidant activities of curcumin (42). Curcumin also has immunomodulatory properties *in vivo*, as shown by COX-2 downregulation and inhibition of pro-inflammatory enzymes, such as LOX-5 and iNOS. It furthermore inhibits the production of important pro-inflammatory cytokines, such as TNF-α and IFN-γ, the latter one by suppressing JAK-STAT- and NF-κ-related signaling cascades (43, 44).

In the current study, the efficacy of curcumin against tachyzoites of *B. besnoiti* is demonstrated *in vitro* for the first time. The natural compound curcumin led to reduce *B. besnoiti* tachyzoite viability, with up to 56% mortality (IC_50_ 5.93 μM) in treated tachyzoites. In line with these findings, studies using lower doses of synthesized compounds such as decoquinate, diclazuril (14), and naptho-quinone buparvaquone (13) also exhibited promising *in vitro* activities against *B. besnoiti* as the ones observed with curcumin. It could be argued that curcumin presented a lower efficacy compared to the mentioned drugs, but it is noteworthy that curcumin is a natural compound and may represent an important alternative measure to control parasitoses (19–21). Moreover, the well-known limitations of poor absorption and bioavailability of curcumin associated with the higher IC_50_ obtained here could hamper the utility of curcumin; however, new research in the field of nanotechnology has shown promising results with nanoparticulate methods (45) and curcumin nanocapsules to be used for drug delivery (46), which might contribute to the improvement of bioavailability of the compound.

So far, the molecular mechanism of curcumin antiprotozoan efficacy is not entirely known. Reddy et al. (47) proposed that arco/endoplasmic reticulum Ca^2+^-ATPase (SERCA) might be targeted since Ca^2+^ levels control a variety of cellular functions in protozoans and further acts as an important second messenger in signaling pathways regulating protein secretion, motility, genetic expression and intracellular development in eukaryotic cells. One of the main cellular sources of Ca^2+^ is the endoplasmic reticulum (ER). Interestingly, *T. gondii* was reported to own a SERCA-like molecule which pumps Ca^2+^ into the ER lumen (48). Moreover, extracellular Ca^2+^ deficiency inhibits tachyzoite invasion of host cells, most probably due to pivotal role in microneme protein secretion (49). Curcumin was reported as a potent inhibitor of SERCA activity at a IC_50_ of 7–15 μM (50), which is in concordance with our data (maximum 8 μM).

Besides Ca^++^-related actions, another target of curcumin might be the cytoskeleton and microtubular composition of *B. besnoiti* tachyzoites. The polar ring (PR) and conoid structures are both situated at the apical edge of the parasite and form part of parasite cytoskeleton. PR is formed by the microtubule-organizing center, whilst the conoid is formed by α- and β-tubulin polymers. These structures are important for parasite gliding motility, tissue migration, and host cell invasion which also dependent on actin filaments with Ca^2+^ being involved (48, 51). In line, curcumin treatments (4 and 8 μM) significantly reduced tachyzoite general motility and invasion capacity *in vitro*. Consequently, infection rates of adequate host cells drastically dropped in curcumin-treated groups. Similar effects have previously been reported for curcumin-treated (6.25 and 12.5 μM) *E. tenella* sporozoites which additionally showed a loss of integrity and viability (19). In addition, *B. besnoiti* tachyzoites revealed enhanced mortality and lost their characteristic banana-shape after curcumin treatment. Thus, curcumin-exposed tachyzoites showed a rather blunt ending at their apical tip and tachyzoite surface appeared irregular. It seems reasonable to assume that the here observed effects of curcumin may be due to alterations of Ca^2+^ influx-dependent biochemical pathways since apicomplexan motility is linked to both, a Ca^++^-dependent actin-myosine-related gliding mechanism and secretion of various proteins enabling successful host cell invasion (52). However, in case of *Giardia intestinalis* stages, curcumin-related anti-parasitic effects were attributed to cytoskeletal impairment. Thus, the distribution and organization of tubulin was altered and its expression decreased in *G. intestinalis* trophozoites (29).

Another potential therapeutic target is the glyoxalase 1, an enzyme of the glyoxalase pathway, a cellular system that eliminates cytotoxic methylglyoxal produced during glycolysis (27). Curcumin was shown to inhibit the enzymatic activity of glyoxalase 1, and consequently to inhibit *in vitro* proliferation of *T. gondii* (27), a very close related parasite to *B. besnoiti*.

Besides general motility, we also analyzed the effects of curcumin on different types of tachyzoite locomotion. Overall, curcumin treatments (8 μM) mainly affected helical gliding and twirling of tachyzoites whilst gliding motility was not altered. Gliding motility allows for movement across cell surfaces and for dissemination within the host and is also a prerequisite for tachyzoite cell invasion, since it provides the force for the active invagination of the host cell membrane. Whilst twirling allows the parasite to position itself to successfully invade a host cell, helical gliding represents a more progressive type of tachyzoite locomotion allowing the parasite to traverse longer distances of 10–200 μm within the host (32), most probably in search of adequate host cells. Here, helical gliding and twirling were significantly reduced. Therefore, alterations of motility caused by higher concentrations of curcumin, were correlated with lower infection rates with the same treatments.

Overall, the current findings proved that curcumin as a natural, plant-derived compound exhibits anticoccidial activity against *B. besnoiti* tachyzoites *in vitro*. Thus, it is reasonable to assume that curcumin/*C. longa* could represent an effective alternative to synthetic chemical compounds for the control of *B. besnoiti* in bovines and maybe also other hosts suffering besnoitiosis (e.g., goats, equids, rabbits, reindeers). However, the lack of a suitable *in vivo* model of besnoitiosis complicates the so needed further *in vivo* experiments to increase the knowledge on pharmacokinetics, bioavailability and pharmacodynamics of curcumin. Nevertheless, the low costs and convenience of curcumin production offers this compound as a rather cheap resource for low-income cattle or other livestock industries worldwide, especially in Africa, South America, and Asia.

## Author Contributions

MC-V, LS, CH, and AT designed the experiments. MC-V and LS performed the experiments. YA-C, GT, MC-V, and LS analyzed and interpreted data. MC-V, LS, CH, and AT wrote and edited the manuscript. All authors approved the final version of the manuscript.

### Conflict of Interest Statement

The authors declare that the research was conducted in the absence of any commercial or financial relationships that could be construed as a potential conflict of interest.
