# Supplementary figures and images for: Antiparasitic Efficacy of Curcumin Against Besnoitia besnoiti Tachyzoites in vitro
Source: Front Vet Sci. 2019 Jan 11;5:333. doi: 10.3389/fvets.2018.00333 (PMC6336690; doi:10.3389/fvets.2018.00333)

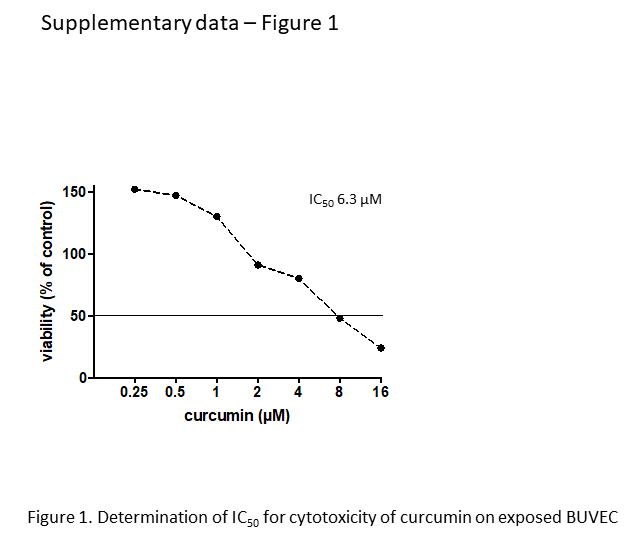

Supplement: Supplementary file 2 [file Image_1.TIF]
